# CX_3_CL1 promotes tumour cell by inducing tyrosine phosphorylation of cortactin in lung cancer

**DOI:** 10.1111/jcmm.15887

**Published:** 2020-11-15

**Authors:** Mengtian Fan, Jinghong Wu, Xian Li, Yingjiu Jiang, Xiaowen Wang, Mengjun Bie, Yaguang Weng, Sicheng Chen, Bin Chen, Liqin An, Menghao Zhang, Gaigai Huang, Mengying Zhu, Qiong Shi

**Affiliations:** ^1^ Ministry of Education Key Laboratory of Diagnostic Medicine School of Laboratory Medicine Chongqing Medical University Chongqing China; ^2^ Department of Pathology The first Affiliated Hospital Chongqing Medical University Chongqing China; ^3^ Cardiothoracic Surgery of the First Affiliated Hospital Chongqing Medical University Chongqing China

**Keywords:** cancer, cortactin, CX_3_CL1, invasion, lung

## Abstract

It has been reported that chemokine CX_3_CL1 can regulate various tumours by binding to its unique receptor CX_3_CR1. However, the effect of CX_3_CL1‐CX_3_CR1 on the lung adenocarcinoma and lung squamous cell carcinoma is still unclear. Here, we showed that CX_3_CL1 can further invasion and migration of lung adenocarcinoma A549 and lung squamous cell carcinoma H520. In addition, Western blot and immunofluorescence test indicated CX_3_CL1 up‐regulated the phosphorylation level of cortactin, which is a marker of cell pseudopodium. Meanwhile, the phosphorylation levels of c‐Src and c‐Abl, which are closely related to the regulation of cortactin phosphorylation, are elevated. Nevertheless, the src/abl inhibitor bosutinib and mutations of cortactin phosphorylation site could inhibit the promotion effect of CX_3_CL1 on invasion and migration of A549 and H520. Moreover, these results of MTT, Hoechst staining and Western blot suggested that CX_3_CL1 had no effect on the proliferation and apoptosis of A549 and H520 in vitro. The effects of CX_3_CL1 were also verified by the subcutaneous tumour formation in nude mice, which showed that it could promote proliferation and invasion of A549 in vivo. In summary, our results indicated that CX_3_CL1 furthered invasion and migration in lung cancer cells partly via activating cortactin, and CX_3_CL1 may be a potential molecule in regulating the migration and invasion of lung cancer.

## 1 INTRODUCTION

Lung cancer is a common malignancy.^1^ Since many patients have had metastasis of bones or brains when receiving treatment for the first time, thus losing the optimal timing of surgical treatment.^2^, ^3^ Current targeted therapies for lung cancer can prolong life to some extent. However, individual differences of patients may limit the sensitivity of oncology drugs. Meanwhile, the use of drugs that target tumours will inevitably lead to drug resistance, ultimately leading to the treatment failure.^4^, ^5^, ^6^, ^7^ Therefore, the development of new diagnostic and therapeutic targets in lung cancer is very important.

Chemokines are a group of small molecule proteins with 8‐10 kD.^8^ By binding to their associated G protein‐coupled receptors, chemokines initiate a series of intracellular reactions, which promote directional migration or chemotactic behaviour in cells.^9^, ^10^ It is reported that tumour cells can secrete chemokines.^11^ Meanwhile, tumour‐associated cells in the tumour microenvironment, such as tumour‐associated macrophages, can also secrete chemokines.^12^ Both sources of chemokines can regulate the progression of tumours.^7^, ^13^ CX_3_CL1 is the only known member of CX_3_C family.^14^ Some studies showed that CX_3_CL1‐CX_3_CR1 can regulate proliferation, invasion, and migration of ovarian cancer,^15^ pancreatic ductal adenocarcinoma,^16^ pancreatic ductal adenocarcinoma prostate cancer ^17^ and other tumours.^18^ In addition, CX_3_CL1‐CX_3_CR1 has been confirmed that it can facilitate metastasis of large cell lung cancer NCI‐H460 cells.^19^ However, the regulation of CX_3_CL1‐CX_3_CR1 on squamous cell carcinoma and adenocarcinomas that are the most common pathological types in non‐small cell lung cancer, as well as the corresponding mechanism, are still unclear. Therefore, further studies on the role of CX_3_CL1‐CX_3_CR1 in lung squamous cell carcinoma and lung adenocarcinoma may provide a new target for clinical diagnosis and therapy.

The cellular pseudopodia marker protein cortactin is mainly localized in the cytoplasm and cell membrane.^20^ It recruits the actin polymerase complex Arp2/3 to participate in the polymerization of actin in the pseudopodia region through the regulation of Src, ERK, PAK, FAK and other kinases.^21^, ^22^, ^23^, ^24^ In addition, the degradation of extracellular matrix by tumour cells can be regulated by the protein by binding to metalloproteinases (MMPs) through the C‐terminal SH3 domain, which promotes tumour cells across the tissue barrier system and accelerates tumour cell metastasis.^25^ At present, it has shown that cortactin plays an important role in the metastasis of various tumour cells such as breast cancer,^26^ head and neck squamous cell cancer,^27^ liver carcinoma,^21^ melanoma,^28^ oesophageal cancer^29^ and bladder cancer.^30^ Phosphorylation of cortactin tyrosine residues is the key for its conformational transformation, recruitment of the Arp2/3 complex and regulation of MMPs.^20^ Studies have shown that cytokines hepatocyte growth factor (HGF), epidermal growth factor (EGF), etc can enhance the phosphorylation of cortactin, promoting the tumour metastasis.^31^, ^32^, ^33^ Due to the universality and the important role of cortactin in tumour cell metastasis, it is believed that exploring the molecular regulation mechanism of CX_3_CL1‐induced phosphorylation of cortactin and the related regulation mechanism of lung cancer metastasis can provide a new theoretical basis for the research of lung cancer metastasis treatment target.

## 2 MATERIALS AND METHODS

### 2.1 Data of specimens

Forty data of lung cancer patients were analysed. Between 2018 and 2019, the Pathology Archive of Chongqing Medical University First Affiliated Hospital (Chongqing, China) provided 40 lung cancer sections with approval of Chongqing Medical University Ethics Committee, and these 40 lung cancer specimens were used to detected p‐cortactin 421 expression level by immunohistochemistry. Informed consent was obtained from all patients.

### 2.2 Immunohistochemistry staining

Paraffin sections of tumour tissues were de‐waxed and rehydrated. After heat‐treating for antigen retrieval with citric acid buffer, the normal goat serum was used to block the sections for 30 minutes. Then, sections were incubated with primary antibody (p‐cortactin 421 1:300, YP0072, ImmunoWay; MMP‐3 1:300, YT4465, ImmunoWay) at 4°C overnight, and then immunohistochemistry kit (PV‐9001, ZSGB‐BIO) was used to analyse the protein expression following standardized protocol. Haematoxylin was used to counterstain sections. Then sections were mounted and coverslipped. The staining degree of sections was assessed by +++: Strong positive; ++: Positive; +: Weak positive; −: Negative.

### 2.3 Cells

Human adenocarcinoma A549 and squamous cell carcinoma H520 cell lines were cultured in complete DMEM (HyClone) supplemented with 10% foetal bovine serum (FBS) and 10 U/mL penicillin‐streptomycin in a 5% CO_2_ incubator at 37°C. 0.5% trypsinase was used to trypsinize cells with a density of 70%‐80%. A549 and H520 cells were incubated with 50 ng/mL or 100 ng/mL recombinant human CX_3_CL1 protein (Sino Biological), and the CX_3_CL1 recombinant protein was generated through eukaryotic source.

### 2.4 siRNA transfection

Mixture of 6 μL/mL ribo*FECT* ^TM^ CP reagent, 60 μL/mL ribo*FECT* ^TM^ CP buffer (1×) and 2.5 μL/mL CX_3_CR1 small interfering RNA (siRNA) was evenly added into the complete DMEM (siCX_3_CR1 group) when the cell density of A549 and H520 reached 50%. However, the same amount of scramble siRNA was used to set up negative control group (NC group). The siRNA, buffer and reagent were purchased from RIBOBIO Co., Ltd.

The following target sequences of siRNA are used:

siCX_3_CR1#1: AGACGCTTAAGCTCTATGA

siCX_3_CR1#2: CCGCAATGTGGAAACAAAT

### 2.5 Plasmid transfection

The mixture of 100 μL/mL medium without FBS and penicillin‐streptomycin, 1μl/ml Lipo2000 and 1 μg/mL cortactin wild‐type plasmid (cortactin WT) or 1 μg/mL cortactin Y421, Y470 and Y486 mutant plasmid (cortactin 3YF) was put at room temperature for 30 minutes before adding into the culture dish evenly. After 4‐6 hours, the medium was changed to new complete medium. The Lipo2000 (Cat.No.11668019) was purchased from Invitrogen.

### 2.6 Western blot

For the Western blot analyses, RIPA buffer containing protease inhibitors and phosphatase inhibitors (Roche) was used to prepare whole‐cell lysates. Twenty micrograms protein of lysates was separated by 10% SDS‐PAGE gels. After SDS‐PAGE electrophoresis, the polyvinylidene difluoride (PVDF) membrane (0.45 μm, Millipore) was put in 5% BSA for blocking at 37°C for 2 hours. After interacting with primary antibodies (CX_3_CR1 1:1000, ab8021, Abcam; MMP‐3 1:1000, YT4465, ImmunoWay; p‐c‐Src 1:1000, YP0077, ImmunoWay; c‐Src 1:1000, YT1139, ImmunoWay; MMP‐9 1:1000, ab76003, Abcam; p‐c‐abl 1:1000, YP0004, ImmunoWay; c‐abl 1:1000, YT0585, ImmunoWay; cortactin 1:500, sc‐55579, santa cruz; p‐cortactin 421 1:1000, YP0072, ImmunoWay) overnight at 4°C, 0.1% TBST was used to wash all membranes for 3 times, 5 minutes each time. IgG antibody of HRP‐antimouse or rabbit (1:3000, ZSGB‐BIO, Beijing, China) was used to combine primary antibodies at 37°C for 1 hour; the membranes were detected by chemiluminescence.

### 2.7 Wound‐healing test

When the cell density reached 80% in the 6‐well plate, the small pipette tip was used to scratch cells. After washing away floating cells by PBS, the medium containing 2% serum was added in each group. Then, the scratch width in same area was observed and photographed under the microscope at 0, 24 and 48 hours. The average width of different observation places and wound‐healing rate was calculated.

### 2.8 Transwell assay

3 × 10^4^ A549 cells or H520 cells were added in 400 μL DMEM containing 2% serum, and the cells suspension was added into the upper chamber. Seven hundred microliters DMEM containing 10% serum with or without CX_3_CL1 was put into the lower well. However, four same groups were used to set up parallel controls. After 24 hours, the transmembrane cells were fixed in 500 μL 4% polyformaldehyde for 20 minutes and 500 μL crystal violet solution was used to stain for 30 minutes. Transmembrane cells were calculated under microscope (×100) after washing and drying. The 50 μL diluted matrigel (Cat.No.356234 Corning) was used in invasion experiment; the diluted ratio of matrigel and culture medium was 1:5.

### 2.9 MTT

2 × 10^3^ H520 or A549 cells were inoculated in 96‐well plate and incubated at 37°C with 5% CO_2_ for ≤4 days. Each well was treated with 10 µL 3‐(4,5‐dimethyl‐2‐thiazolyl)‐2,5‐diphenyl‐2‐H‐tetrazolium bromide (MTT) (5 mg/mL) at 37°C for 4 hours. Medium of each well was removed, and 200µl DMSO was used to lyse MTT for 30 minutes. The viability of A549 and H520 was measured by ELISA plate reader (BioTek Instruments, Inc) at 492 nm.

### 2.10 Crystal violet staining

A549 and H520 cells were inoculated into 24‐well plate and cultured for 48 hours. The medium was removed, and 500 µL 4% paraformaldehyde was added in each well for 30min; then, cells were dyed by 500 µL 1% crystal violet solution for 30 minutes.

### 2.11 Hoechst staining

H520 or A549 cells were inoculated in 6‐well plate; cells were cultured for 48 hours. 4% paraformaldehyde was used to fix cells and 10 ng/mL Hoechst 33 258 solution (Solarbio, #C0021) was used to stain cells, and the increased condensation of chromatin was observed in apoptotic cells. Apoptotic cells were captured with inverted fluorescence microscope (×100).

### 2.12 Immunofluorescent staining

A549 and H520 cells were inoculated onto cover slips in a 24‐well plate overnight. After adding with 100 ng/mL CX_3_CL1 at different time, 4% paraformaldehyde was used to fix cells for 10 minutes. Then, they were permeabilized with 0.1% Triton X‐100 for 10 minutes and blocked in 5% BSA at room temperature for 30 minutes. After incubating with a primary antibody at 4°C overnight, the cover slips of each group were incubated with specific secondary antibodies for 1 hour at room temperature. Next, cells of cover slips were washed by PBS for three times and were stained with 4',6‐diamidino‐2‐phenylindole (DAPI) for 5 minutes. Confocal laser scanning microscope (Thermo Fisher Scientific) was used to detect immunofluorescent staining.

### 2.13 In vivo experiment

The 14 specific pathogen‐free (SPF) male BALB/c nude mice (6 weeks old) were purchased from HFK BIOSCIENCE Co., Ltd (Beijing, China). Fourteen nude mice were divided into two groups randomly (CX_3_CL1 group: n = 8; Blank group: n = 6). A549 cells (5 × 10^6^) were suspended in 100 μL PBS and then inoculated into the subcutaneous region of the right leg of nude mice, the 500 ng CX_3_CL1 was injected every 3 days. These tumours were taken out 3 weeks after tumour implantation. The tumour volume was calculated as: V = a×b^2^ × π/6 (a: volumes of the largest; b: volumes of smallest). The experimental procedures ensured the safety of practitioners in laboratory animal projects and conformed to human ethical standards and international practices and approved by the Animal Experimental Ethics Committee of Chongqing Medical University. Animals were fed in a standard laboratory with controlled temperature (22 ± 1), humidity (65%‐70%) and a 12h:12h light‐dark cycle. These animals were free to get food and water. Isoflurane inhalation (1.5%‐2%) was used to anaesthetize mice, and then, these mice were euthanized by cervical dislocation in the end of study. All animal experiments conform to the guidelines from Directive 2010/63/EU of the European Parliament on the protection of animals used for scientific purposes.

### 2.14 Statistical analysis

All experimental results were analysed by SPSS 16.0 statistical software. The difference of multiple group was analysed using the one‐way ANOVA. The difference between groups was quantitated by *t* test or Bonferroni‐corrected *t* test. The statistical significance of the difference was set at *P* < .05.

## 3 RESULTS

### 3.1 The effect of CX_3_CL1 on the proliferation and apoptosis of A549 and H520 cells

**Figure 1:**
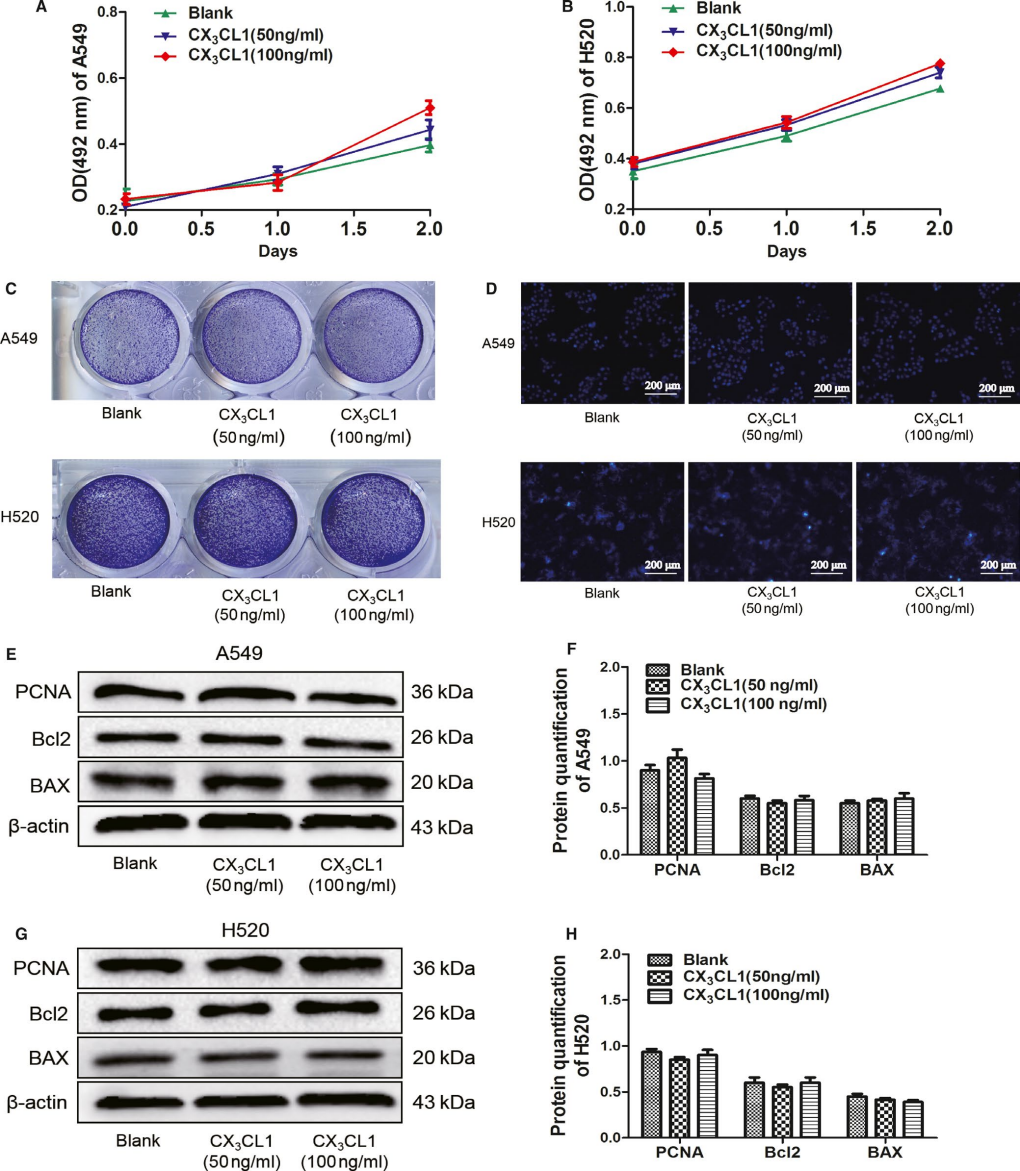
The role of CX_3_CL1 on proliferation and apoptosis in A549 and H520 cells. A, The proliferation ability of A549 cells after adding with 50 ng/mL or 100 ng/mL CX_3_CL1 was detected by MTT (*P* = ns). B, The proliferation ability of H520 cells after adding with 50 ng/mL or 100 ng/mL CX_3_CL1 was detected by MTT (*P* = ns). C, The cell number of A549 (upper) and H520 (lower) cells after adding with 50 ng/mL or 100 ng/mL CX_3_CL1 was detected by crystal violet staining. D, The apoptosis ability of A549 (upper) and H520 (lower) cells after adding with 50 ng/mL or 100 ng/mL CX_3_CL1 was detected by Hoechst staining. E, Western blot was used to detect PCNA, Bcl2 and BAX expression in A549 cells after adding with different concentration of CX_3_CL1. F, Quantitation of PCNA, Bcl2 and BAX by densitometry analysis (*P* = ns). G, Western blot for detecting expression of PCNA, Bcl2 and BAX in H520 cells after adding with different concentration of CX_3_CL1. H, Quantitation of PCNA, Bcl2 and BAX by densitometry analysis (*P* = ns)

To investigate the effect of CX_3_CL1 on the proliferation of A549 and H520 cells, A549 and H520 cells were treated with 50 ng/ml or 100 ng/ml CX_3_CL1 protein. MTT experiment showed that the OD values had no significant difference in each group on day 0, day 1 and day 3 (Figure 1A,B). The results of crystal violet staining indicated that there was no significant difference in the number of A549 and H520 cells in each group (Figure 1C). It is consistent with the results that protein level of PCNA showed no significant difference in each group (Figure 1E‐H). These results suggested that 50 ng/mL or 100 ng/mL CX_3_CL1 has no obvious effect on the proliferation of lung cancer cells. Hoechst staining was used to study the effect of CX_3_CL1 on the apoptosis of A549 and H520 cells. It showed that there were no typical apoptotic changes of the nuclei of A549 and H520 cells in each group (Figure 1D). It is consistent with the results that the protein levels of Bcl2 and BAX showed no significant difference in each group (Figure 1E‐H).

### 3.2 The effect of CX_3_CL1 on invasion and migration of A549 and H520 cells

**Figure 2:**
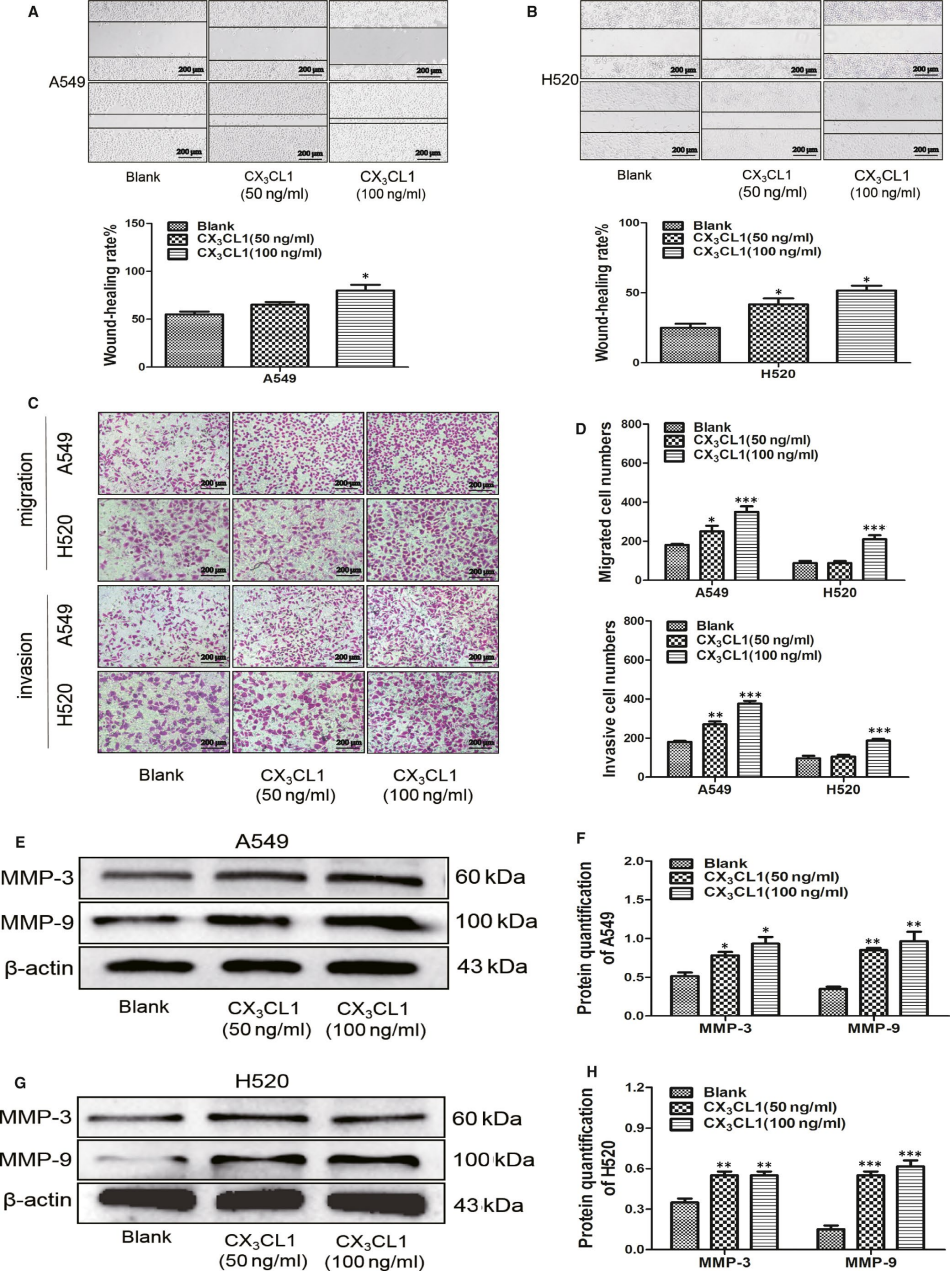
The effect of CX_3_CL1 on invasion and migration of A549 and H520 cells. A, Migrated ability of A549 after adding with 50 ng/mL or 100 ng/mL CX_3_CL1 was verified by wound‐healing test (upper), and the statistical result was shown ( lower, ^*^ *P < *.05 vs Blank group). B, Migration ability at H520 after adding with 50 ng/mL or 100 ng/mL CX_3_CL1 was performed by wound‐healing test (upper), and the statistical result was shown (lower, ^*^ *P < *.05 vs Blank group). C, Invasion and migration abilities were analysed by transwell with or without matrigel at A549 and H520 cells after adding with different concentration of CX_3_CL1. D, The statistical results of transwell without matrigel (upper) and transwell with matrigel (lower) were shown (^**^ *P < *.005 ^***^ *P < *.001 vs Blank group). E, Protein levels of MMP‐3 and MMP‐9 were detected by Western blot in A549 after adding with different concentration of CX_3_CL1. F, Quantitation of MMP‐3 and MMP‐9 by densitometry analysis (^*^ *P < *.05 ^**^ *P < *.005 vs Blank group). G, Western blot was used to detected protein levels of MMP‐3 and MMP‐9 in H520 cells after adding with different concentration of CX_3_CL1. H, Quantitation of MMP‐3 and MMP‐9 by densitometry analysis (^**^ *P < *.005 ^***^ *P < *.001 vs Blank group)

**Figure 3:**
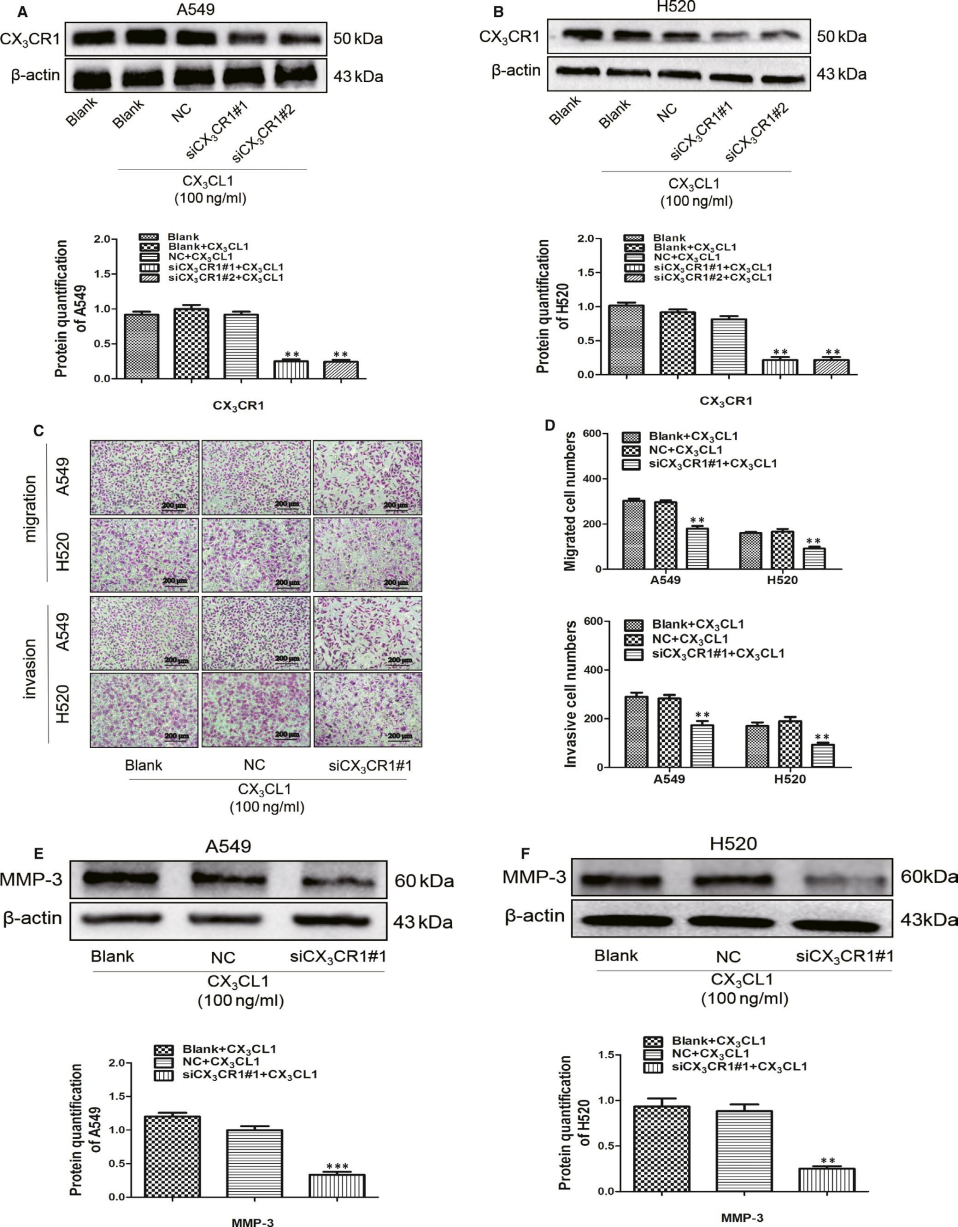
The effect of CX_3_CL1 on migration and invasion of A549 and H520 cells after knocking down CX_3_CR1. A, The expression of CX_3_CR1 was detected by Western blot in A549 cells after knocking down CX_3_CR1 (upper) and quantitation of CX_3_CR1 by densitometry analysis (lower, ^**^ *P < *.005 vs Negative control group). B, The protein level of CX_3_CR1 in H520 cells was detected by Western blot after knocking down CX_3_CR1 (upper) and quantitation of CX_3_CR1 by densitometry analysis (lower, ^**^ *P < *.005 vs Negative control group). C, The invasion and migration abilities were analysed by transwell with or without matrigel at A549 and H520 cells after knocking down CX_3_CR1. D, The statistical results of transwell without matrigel (upper) and transwell with matrigel (lower) were shown (^**^ *P < *.005 vs Negative control + 100 ng/mL CX_3_CL1 group). E, The protein level of MMP‐3 was detected by Western blot in A549 cells after knocking down CX_3_CR1 (upper) and quantitation of MMP‐3 by densitometry analysis (lower, ^***^ *P < *.001 vs Negative control + 100 ng/mL CX_3_CL1 group). F, Expression of MMP‐3 in H520 cells was detected by Western blot after knocking down CX_3_CR1 (upper) and quantitation of MMP‐3 by densitometry analysis (lower, ^**^ *P < *.005 vs Negative control + 100 ng/mL CX_3_CL1 group)

Wound‐healing test verified that the migrated ability of H520 or A549 cells was promoted by adding CX_3_CL1 (Figure 2A,B). Transwell test without matrix was applied to further verify the effect of CX_3_CL1 on the migration abilities of A549 and H520. It revealed that the transmembrane numbers of A549 and H520 cells in CX_3_CL1 group were higher than that in control group (Figure 2C,D). The effect of CX_3_CL1 on invasion abilities of A549 and H520 cells was also verified by the transwell test with matrix. The results showed that the transmembrane numbers of A549 and H520 cells in CX_3_CL1 group were higher than that in control group (Figure 2C,D). It is consistent with the results that the expression of matrix metalloproteinase MMP‐3 and MMP‐9 was up‐regulated in A549 and H520 cells by adding with CX_3_CL1 (Figure 2E‐H). To clarify the effect of CX_3_CL1 on lung cancer cell migration (Figure 3A,B). Transwell test without matrix revealed that the transmembrane numbers of A549 and H520 cells were lower in CX_3_CR1 knockdown group than that in control group (Figure 3C,D). Meanwhile, transwell test with matrix indicated that the transmembrane numbers of A549 and H520 cells were lower in CX_3_CR1 knockdown group than that in control group (Figure 3C,D), and the protein level of MMP‐3 was down‐regulated in A549 and H520 cells by transfecting siCX_3_CR1 (Figure 3E,F).

### 3.3 The effect of CX_3_CL1 on A549 cell in vivo

**Figure 4:**
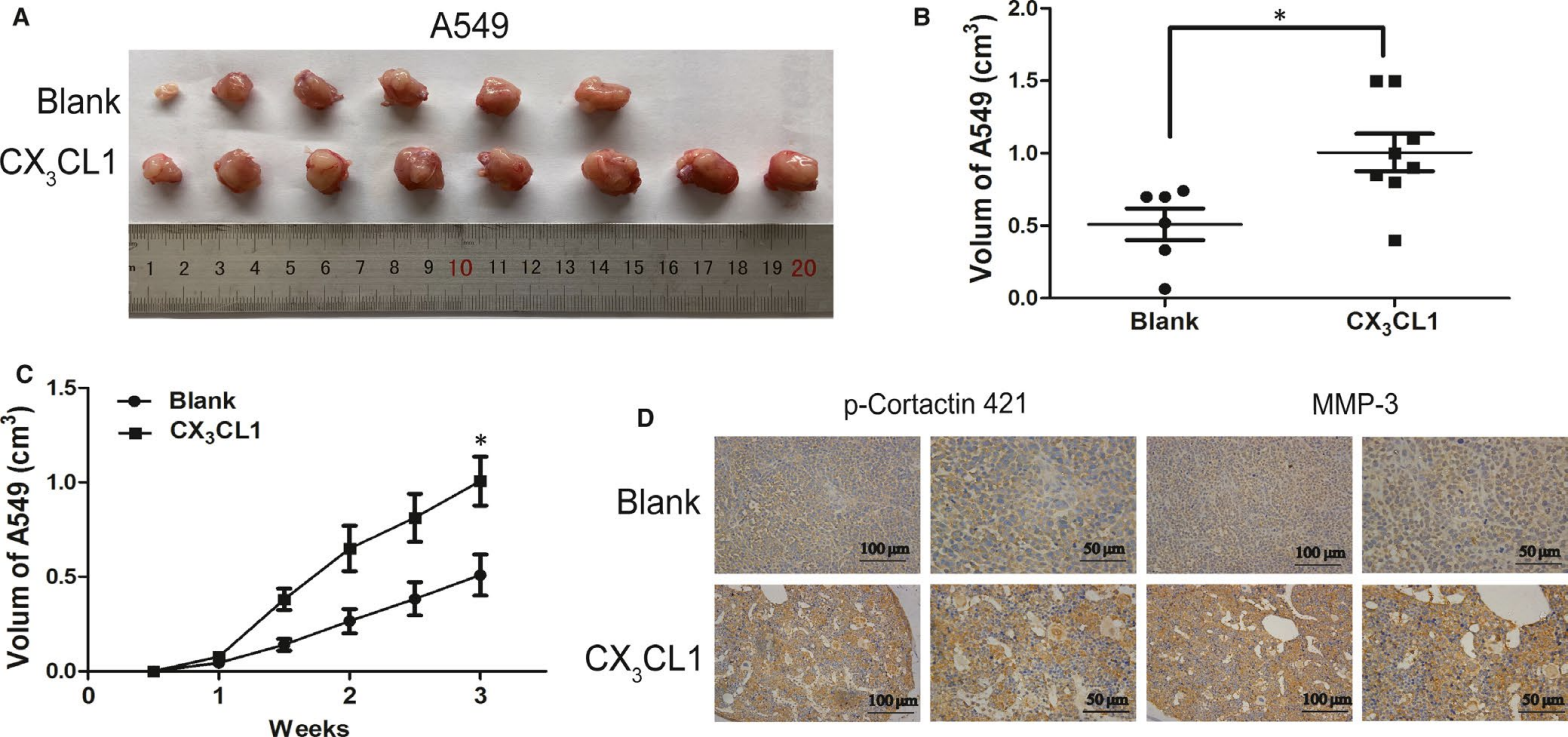
The effect of CX_3_CL1 on A549 cells in vivo. A, The A549 tumours were took out from nude mice for 3 wk after tumour implantation (Blank group: n = 6; CX_3_CL1 group: n = 8). B, The tumour growth curves were shown (^*^ *P < *.05 vs Blank group). C, The statistical result of tumour volume was shown (^*^ *P < *.05 vs Blank group). D, The expression of p‐cortactin 421 and MMP‐3 in tumours of nude mice were detected by immunohistochemistry

To demonstrate the effect of CX_3_CL1 on lung cancer in vivo, A549 cells with the number of 5 × 10^6^ together with 500 ng CX_3_CL1 were implanted into the nude mice to construct the xenograft model. It is observed in the in vivo experiments that the tumour formation and growth was increased by adding with CX_3_CL1 compared to control mice (Figure 4A‐C), and immunohistochemistry results showed that p‐cortactin 421 and MMP‐3 were up‐regulated in CX_3_CL1 group (Figure 4D). In addition, immunohistochemistry results showed that PCNA was up‐regulated in CX_3_CL1 group (Figure S1).

### 3.4 The effect of CX_3_CL1 on phosphorylation of cortactin

**Figure 5:**
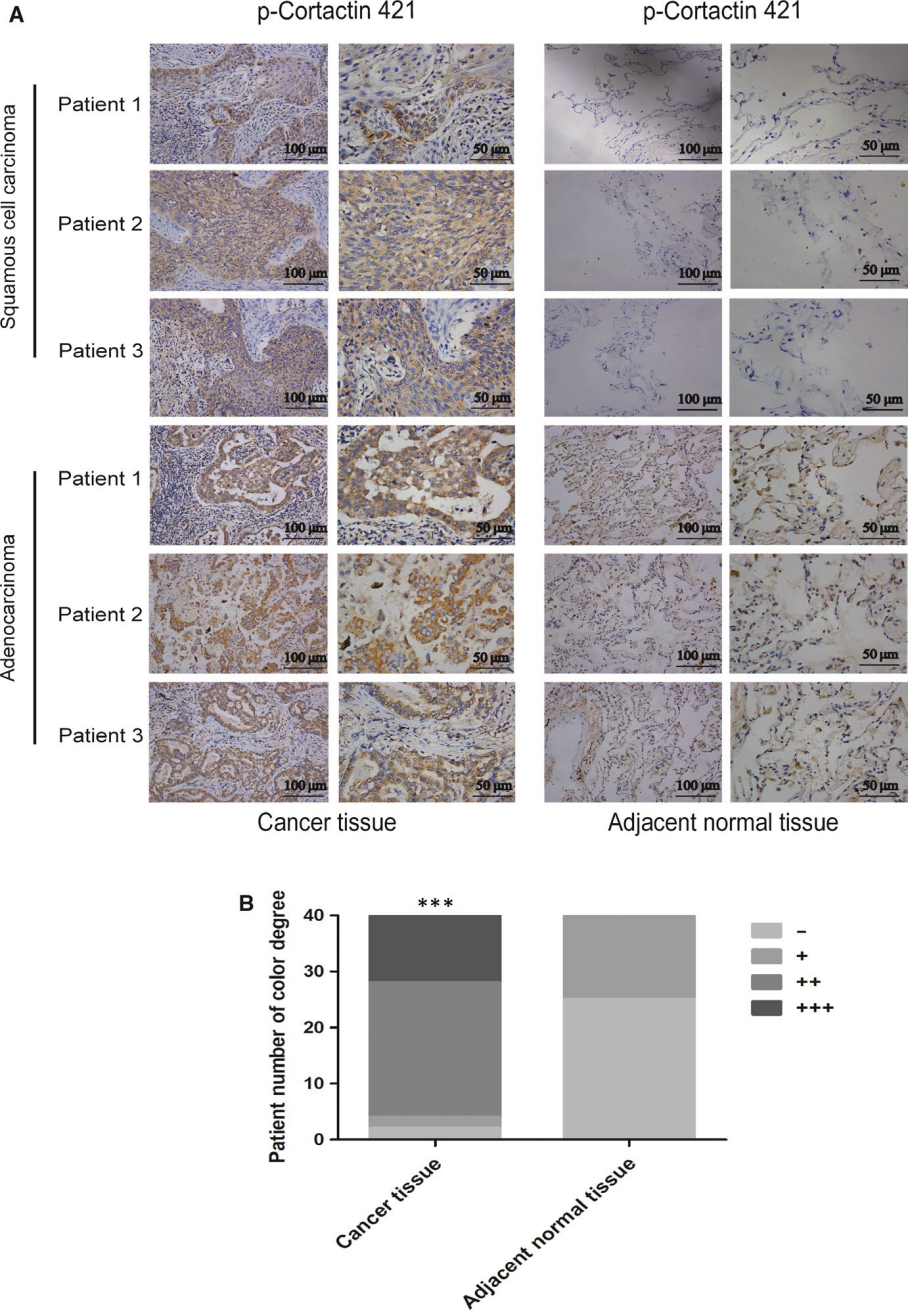
The expression of p‐cortactin 421 in lung cancer patients. A, The expression of p‐cortactin 421 in lung cancer patients was detected by immunohistochemistry. B, Quantitation of immunohistochemistry staining degree was shown (^***^ *P < *.001 vs Adjacent normal tissue)

**Figure 6:**
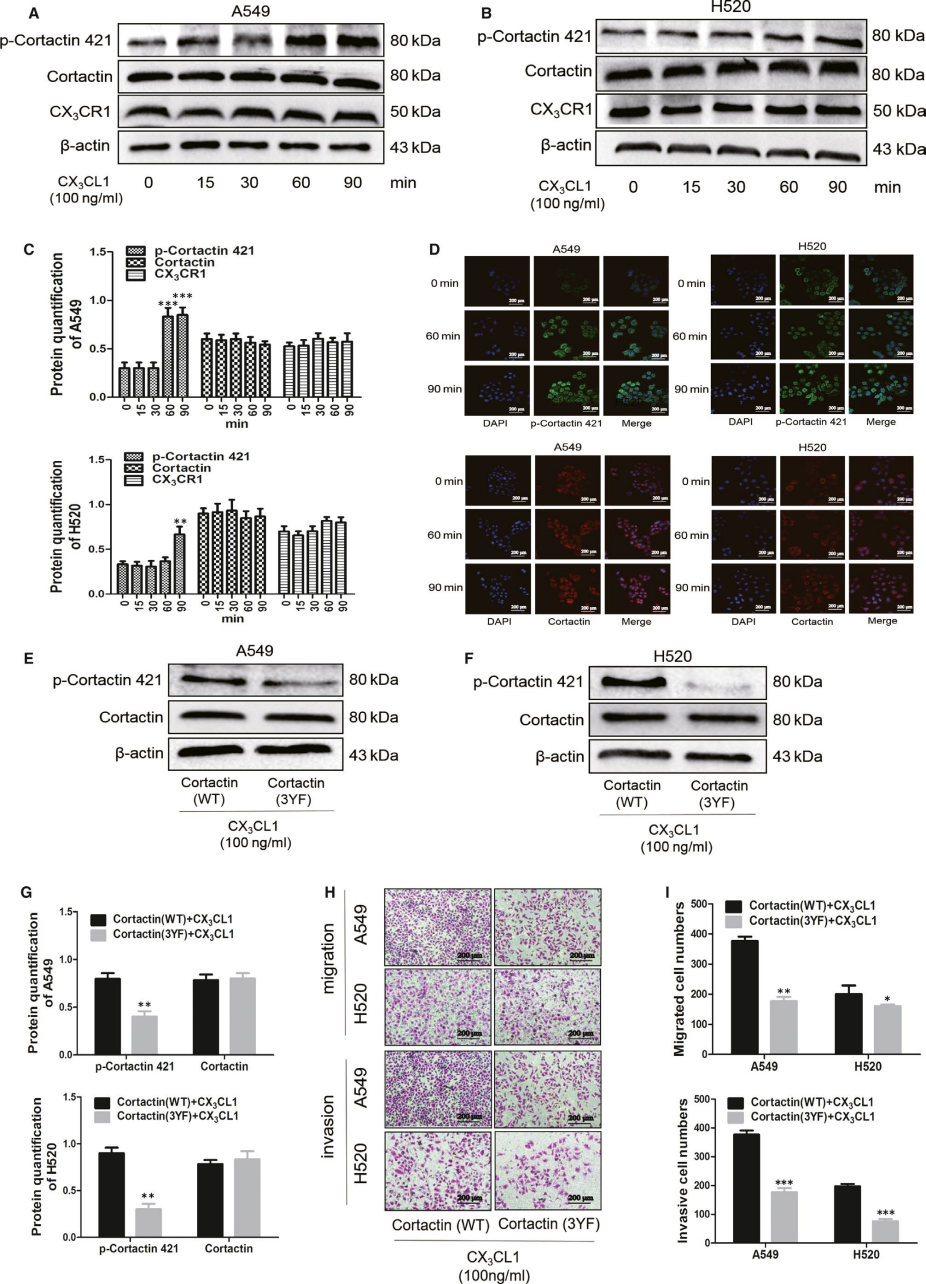
The effect of CX_3_CL1 on phosphorylation of cortactin. A, The protein levels of p‐cortactin 421, cortactin and CX_3_CR1 were detected by Western blot in A549 cells after adding with 100 ng/mL CX_3_CL1 at different time. B, The protein levels of p‐cortactin 421, cortactin and CX_3_CR1 in H520 cells were detected by Western blot after adding with 100 ng/mL CX_3_CL1 at different time. C, Densitometry analysis quantify the expression of p‐cortactin 421, cortactin and CX_3_CR1 in A549 cells (upper, ^***^ *P < *.001 vs 0 min group) and H520 cells (lower, ^**^ *P < *.005 vs 0 min group). D, The expression of p‐cortactin 421 and cortactin were detected by immunofluorescence after adding with 100 ng/mL CX_3_CL1 at 0, 60 and 90 min. E, Western blot was used to detected the protein levels of p‐cortactin 421 and cortactin in A549 cells after transfecting cortactin (WT) or cortactin (3YF) plasmid. F, Expression of p‐cortactin 421 and cortactin were detected in H520 cells after transfecting cortactin (WT) or cortactin (3YF) plasmid. G, Quantitation of p‐cortactin 421 and cortactin in A549 cells (upper, ^**^ *P < *.005 vs cortactin (WT)+100 ng/mL CX_3_CL1 group) and H520 cells (lower, ^**^ *P < *.005 vs cortactin (WT)+100 ng/mL CX_3_CL1 group). H, The invasion and migration abilities were analysed by transwell with or without matrigel at A549 and H520 cells after transfecting cortactin (WT) or cortactin (3YF) plasmid. I, The statistical results of transwell without matrigel (upper) and transwell with matrigel (lower) were shown (^*^ *P < *.05 ^**^ *P < *.005 ^***^ *P < *.001 vs cortactin (WT)+100 ng/mL CX_3_CL1 group)

cortactin acts as a marker for cellular pseudopods, whereas its activation is an important marker of tumour metastasis.^20^ Immunohistochemistry results showed that in patients with lung adenocarcinoma and lung squamous cell carcinoma, the expression level of p‐cortactin 421 in lung cancer tissues was significantly higher than that in adjacent tissues (Figure 5A,B). To further investigate the role of cortactin in the regulation of A549 and H520 metastasis during CX_3_CL1, Western blot was performed. The result exhibited that the protein level of p‐cortactin 421 in A549 cells was up‐regulated at 60min and maintained to 90 minutes in CX_3_CL1 group (Figure 6A,C), and the protein level of p‐cortactin 421 in H520 cells was up‐regulated at 90 minutes treated with CX_3_CL1 (Figure 6B,C). However, the protein levels of cortactin and CX_3_CR1 showed no significant difference in each group (Figure 6A‐C). In addition, it is observed in the immunofluorescence experiments that CX_3_CL1 promoted the expression of p‐cortactin 421 in A549 and H520 cells, whereas it had no effect on the expression of cortactin (Figure 6D). To explore the role of cortactin in the regulation of migration and invasion in A549 and H520 by CX_3_CL1, we selected the WT and mutant plasmids of cortactin. At the same concentration of CX_3_CL1, the transwell without matrigel showed that the number of transmembrane cells in the cortactin mutant group was significantly reduced compared to the control (Figure 6H,I). Similarly, the transwell with matrigel indicated that transmembrane cells in the cortactin mutant group were significantly reduced compared to the control (Figure 6H,I). The results of Western blot suggested that the level of p‐cortactin 421 in the cortactin mutant group was lower under CX_3_CL1 treatment. Similarly, no significant change of cortactin was observed (Figure 6E‐G).

### 3.5 The effect of CX_3_CL1 on phosphorylation of cortactin via c‐Src and c‐Abl pathway

**Figure 7:**
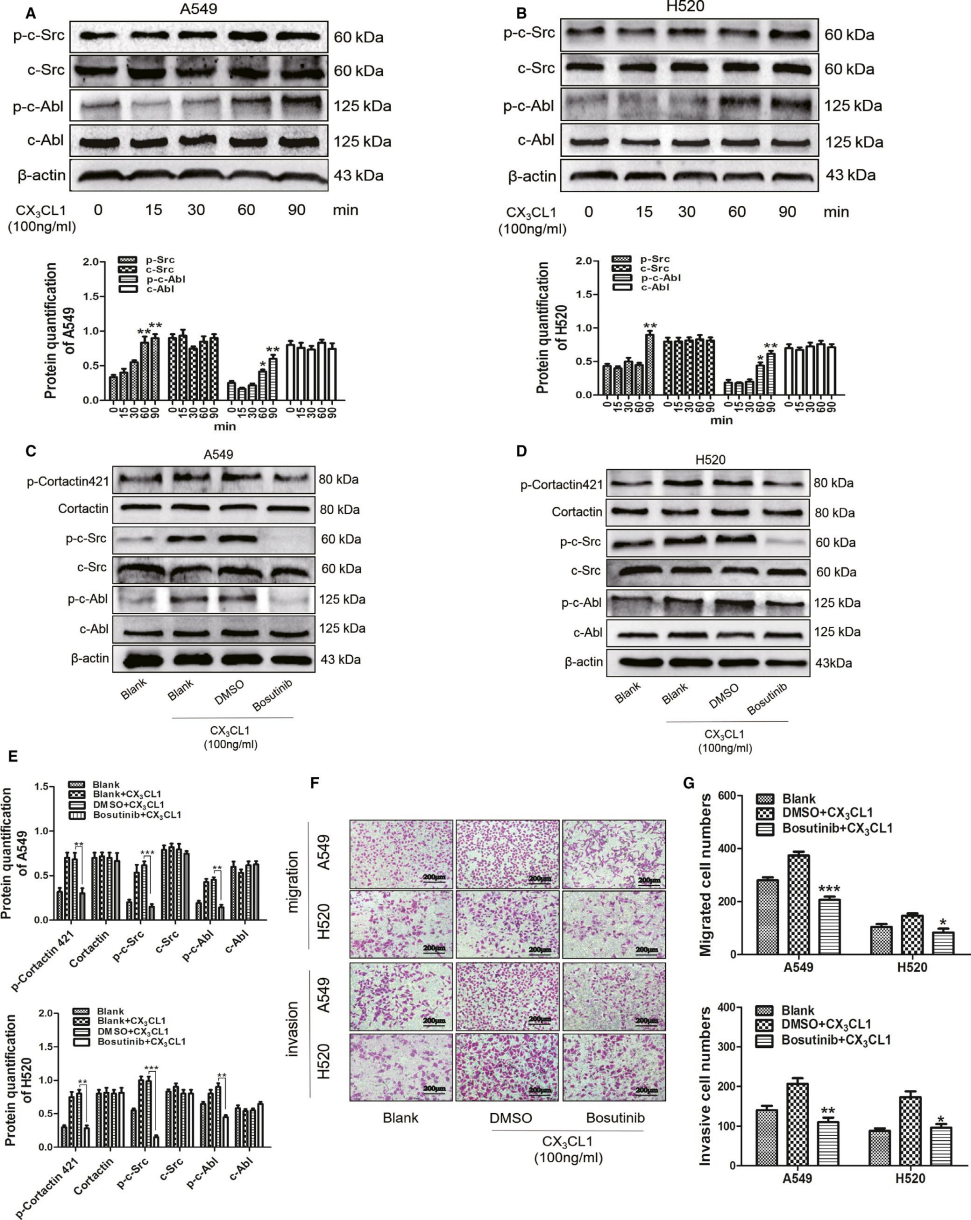
The effect of CX_3_CL1 on phosphorylation of cortactin via c‐Src and c‐Abl pathway. A, The protein levels were detected by Western blot in A549 cells after adding with 100 ng/mL CX_3_CL1 at different time (upper) and densitometry analysis quantify the expression in A549 cells (lower, ^*^ *P < *.05 ^**^ *P < *.005 vs 0 min group). B, The protein expression was detected in H520 cells after adding with 100 ng/mL CX_3_CL1 at different time (upper) and densitometry analysis quantify the expression (lower, ^*^ *P < *.05 ^**^ *P < *.005 vs 0 min group). C, The protein expression was detected by Western blot in A549 cells after adding with bosutinib. D, The protein levels were detected by Western blot in H520 cells after adding with bosutinib. E, Densitometry analysis quantify the expression of p‐cortactin 421, cortactin, p‐c‐Src, p‐c‐Abl, c‐Src and c‐Abl in A549 cells (upper, ^**^ *P < *.005 ^***^ *P < *.001 vs DMSO + 100 ng/mL CX_3_CL1 group) and H520 cells (lower, ^**^ *P < *.005 ^***^ *P < *.001 vs DMSO + 100 ng/mL CX_3_CL1 group). F, The invasion and migration abilities were analysed by transwell with or without matrigel at A549 and H520 cells after adding with bosutinib. G, The statistical results of transwell without matrigel (upper) and transwell with matrigel (lower) were shown (^*^ *P < *.05 ^**^ *P < *.005 ^***^ *P < *.001 vs DMSO + 100 ng/mL CX_3_CL1 group)

It has been reported that c‐Src and c‐Abl are important kinases that regulate the phosphorylation of cortactin 421.^19^ Western blot showed that the protein levels of p‐c‐Src and p‐c‐Abl were up‐regulated in CX_3_CL1 group compared with control (Figure 7A,B). However, after adding 10 nmol/L bosutinib, which was a specific inhibitor of c‐Src and c‐Abl pathway, the protein levels of p‐c‐Src, p‐c‐Abl, and p‐cortactin 421 were inhibited (Figure 7C‐E). In contrast, the protein levels of c‐Src, c‐Abl and cortactin showed no significant difference in each group (Figure 7A‐E). To verify the effect of bosutinib on migration ability of A549 and H520 cells, transwell without matrigel was carried out. The result showed that the number of transmembrane cell in the bosutinib group was significantly lower than that in the control group after CX_3_CL1 treatment (Figure 7F,G). Similarly, the results of transwell with matrigel showed that the number of transmembrane cell in the bosutinib group was dramatically reduced compared to control (Figure 7F,G).

## 4 DISCUSSION

Lung cancer is originated from the bronchial epithelium, in which non‐small cell lung cancer accounts for 80%.^34^ Only a small proportion of patients can be diagnosed at an early stage in lung cancer.^35^ The main cause for the treatment failure and patient death is the cancer migration and invasion, which occurs in various organs in the advanced stage of cancers^36^. Tumour metastasis is a complex process involving a variety of biological behaviours such as decreased adhesion between tumour cells, extracellular matrix degradation, enhanced tumour cell invasion and tumour angiogenesis.^37^ Therefore, exploring the mechanism of lung cancer migration and invasion will provide new ideas for the diagnosis and treatment of lung cancer.

Recently, many studies have suggested that chemokine axes are involved in regulating tumour growth, migration and invasion. Chemokines and their receptors can be classified into C, CC, CXC and CX_3_C according to the number and interval of N‐terminal cysteine.^38^ Among them, the chemokine CX_3_CL1 belongs to the CX_3_C family. Currently, the only found natural receptor for CX_3_CL1 is the chemokine CX_3_CR1. The chemokine axis CX_3_CL1‐CX_3_CR1 has demonstrated the ability to promote migration and invasion of various tumours. Some studies have shown that by activating PI3K/Akt/NF‐KB, CX_3_CL1‐CX_3_CR1 can promote the metastasis of osteosarcoma.^39^ Other researchers have found that CX_3_CL1‐CX_3_CR1 can regulate the invasion, proliferation and migration of prostate cancer via PI3K/AKT pathway.^40^ In addition, CX_3_CL1‐CX_3_CR1 was observed to regulate the development of breast cancer by activating the MAPK/ERK signalling pathway.^16^ Our group has confirmed that 100ng/ml of CX_3_CL1 can up‐regulate migrated and invasive abilities of lung cancer A549 cells and H520 cells. After knocking down the unique receptor of CX_3_CL1, that is CX_3_CR1, the ability of CX_3_CL1 to promote A549 and H520 cells was inhibited. CX_3_CL1 and CX_3_CR1, as the only members of the CX_3_C family, only bind to each other, both of which are not shared by other chemokines. Since CX_3_CL1 is an exocrine small molecule protein that can be detected in serum, we will further collect serum samples from lung cancer patients in the next experiment. The relationship between the expression of CX_3_CL1 and the pathological type and malignant degree of lung cancer will be explored, aiming to provide some ideas for CX_3_CL1 as a clinically diagnostic and prognostic indicator.

Tumour migration or invasion is a complex process, in which cell deformation and movement are the basis for the process.^41^, ^42^ It has been found in those studies that cortactin exhibited a high expression trend in various tumours and promoted tumour metastasis. cortactin has multiple kinase regulatory sites, in which phosphorylation of tyrosine residues is the key to its conformational transformation, recruitment of Arp2/3 complexes and regulation of MMPs. Phosphorylated Y421 and Y466 interact with SH2 and SH3 domain‐rich proteins, like Src, N‐WASP, Nck1 and FAK. The binding of these proteins further stabilizes the conformation of cortactin and recruits more Arp2/3 complexes, thereby promoting the polymerization of actin in the pseudopod and maintaining the formation and dynamic changes of pseudopods in tumour cells.^20^, ^21^, ^43^, ^44^ Furthermore, a high expression of p‐cortactin 421 in lung adenocarcinoma and lung squamous cell carcinoma compared with adjacent tissues was observed in the experiment. However, in vitro experiments showed that the expression level of the phosphorylated form of cortactin was increased, whereas the expression level of cortactin was no change after adding with CX_3_CL1 at a concentration of 100 ng/mL for 90 minutes. After the phosphorylation sites of cortactin were mutated, the wound‐healing experiment and the transwell experiment showed that the ability of CX_3_CL1 to promote cell migration and invasion was significantly inhibited. It implies that the migration and invasion of A549 and H520 are promoted by CX_3_CL1 through the regulation of the phosphorylation of cortactin, rather than through increasing the expression of cortactin. In addition, studies have confirmed that cortactin is highly expressed in many tumours. We observed in the experiment that the increased expression of p‐cortactin in lung cancer tissue may be due to the increased expression of cortactin, so we need more research to confirm the effect of CX_3_CL1 on the phosphorylation of cortactin in vivo. In the mechanism exploration, the c‐Src and c‐Abl signalling pathways were selected as the objects. The results showed that p‐c‐Abl and p‐c‐Src were increased by CX_3_CL1 with the concentration of 100 ng/mL. On the other hand, bosutinib, as the c‐Abl and c‐Src inhibitor, inhibited the activation of p‐c‐Src and p‐c‐Abl by CX_3_CL1. In addition to the phosphorylation of tyrosine sites, studies have confirmed that phosphorylation of serine/threonine sites is also an important pathway for cortactin to function. MAPK/ERK, PAK and other signalling pathways have been found with the ability of regulating the phosphorylation of cortactin 405 and 418 serine sites.^45^, ^46^ At the same time, CX_3_CL1 has been reported to regulate the metastasis of various tumours through MAPK/ERK and PI3K/AKT signalling pathways.^16^, ^40^ Therefore, in the next experiment, we will further investigate the regulation mechanism of the metastasis of lung cancer by verifying whether CX_3_CL1 regulates the phosphorylation of cortactin through MAPK/ERK and PI3K/AKT signalling pathways.

CX_3_CL1 is divided into two types: soluble CX_3_CL1 and membrane‐bound CX_3_CL1. Soluble CX_3_CL1 mainly binds to cells that expressing CX_3_CR1 to regulate cell migration. Membrane‐bound receptor type is mainly expressed on surface of cell membrane and participates in regulating adhesion between cells.^47^ In prostate cancer studies, osteoblasts have been found to express soluble and membrane‐bound CX_3_CL1, which promotes prostate cancer to metastasize and colonize bone tissue.^40^ Our previous study also confirmed that bone marrow stromal cells HS5 could accelerate invasive and migrated abilities of lung adenocarcinoma A549 cells. In this study, we focused on exploring the effect of soluble CX_3_CL1 in regulating lung cancer A549 and H520 cells. In the next experiment, we will further explore the effect of membrane‐bound CX_3_CL1 on lung cancer to verify whether it is related to the colonization of lung cancer cells in the metastatic tissue.

In vivo experiments, we observed that 500 ng of CX_3_CL1 promoted the formation and growth of A549. However, in vitro experiments, the same concentration of CX_3_CL1 had no effect on proliferation or apoptosis of A549 and H520, but furthered migration and invasion of A549 and H520. Thus, the same experimental model leads to different results of in vivo and in vitro experiments. The happening and development of tumours is a compound process, which is the outcome of the interactions between tumour cells and their microenvironment.^48^ Although nude mice lack T cells, immune cells such as B cells, macrophages and granulocytes are still expressed. Studies have demonstrated that in addition to regulating the biological functions of tumour cells, the chemokine axis CX_3_CL1‐CX_3_CR1 regulates NK cells, T cells, macrophages, etc in the tumour microenvironment. For instance, CX_3_CL1‐CX_3_CR1 is highly expressed on the surface of non‐classical macrophages (M2 macrophages) in breast cancer models.^49^, ^50^, ^51^, ^52^ Therefore, we speculate that besides promoting the invasion directly by affecting on tumour cells, in vivo injection of CX_3_CL1 may also be involved in the recruitment of M2 macrophages with high expression of CX_3_CR1. The entered M2 macrophages in lung cancer tissues further promote the proliferation and metastasis of lung cancer. In addition, some studies have confirmed that CX_3_CL1 significantly enhances NK cell cytotoxicity, IFN‐γ expression and secretion to inhibit neuroblastoma proliferation and metastasis.^53^ Based on the preliminary understanding of the role of CX_3_CL1‐CX_3_CR1 on lung cancer cells obtained in this study, we will further explore the effect of CX_3_CL1‐CX_3_CR1 on the development of lung cancer from the aspect of tumour microenvironment.

To sum up, our research suggested that CX_3_CL1 could promote invasion and migration of lung cancer A549 and H520 cells by up‐regulating the phosphorylation of cortactin, which might be a potential target of clinical diagnosis and therapy.

## 5 CONCLUSIONS

The current study demonstrates that CX_3_CL1 promotes the phosphorylation of cortactin via c‐Src and c‐Abl signalling pathways, and further promotes the migration and invasion of lung adenocarcinoma A549 cells and lung squamous cell carcinoma H520 cells. The promotion effect of CX_3_CL1 can be inhibited by the Src/Abl inhibitor bosutinib. The results of this study may provide new ideas for the clinical diagnosis and treatment of lung cancer.

## CONFLICT OF INTEREST

The authors declare that they have no conflicts of interest.

## AUTHOR CONTRIBUTIONS

**Mengtian Fan:** Data curation (equal); Writing‐original draft (equal). **Jinghong Wu:** Data curation (equal); Writing‐original draft (equal). **Xian Li:** Resources (supporting). **Yingjiu Jiang:** Resources (supporting). **Xiaowen Wang:** Methodology (supporting). **Mengjun Bie:** Methodology (supporting). **Yaguang Weng:** Formal analysis (supporting). **Sicheng Chen:** Software (supporting). **Bin Chen:** Software (supporting). **Liqin An:** Software (supporting). **Menghao Zhang:** Software (supporting). **Gaigai Huang:** Writing‐original draft (supporting). **Mengying Zhu:** Writing‐original draft (supporting). **Qiong Shi:** Formal analysis (lead); Funding acquisition (lead); Supervision (lead).

## Supporting information

Figure S1

## Data Availability

The data sets generated during and/or analysed during the current study are available from the corresponding author on reasonable request.
