# Supplementary figures and images for: CX3CL1 promotes tumour cell by inducing tyrosine phosphorylation of cortactin in lung cancer
Source: J Cell Mol Med. 2020 Nov 15;25(1):132–46. doi: 10.1111/jcmm.15887 (PMC7810942; doi:10.1111/jcmm.15887)

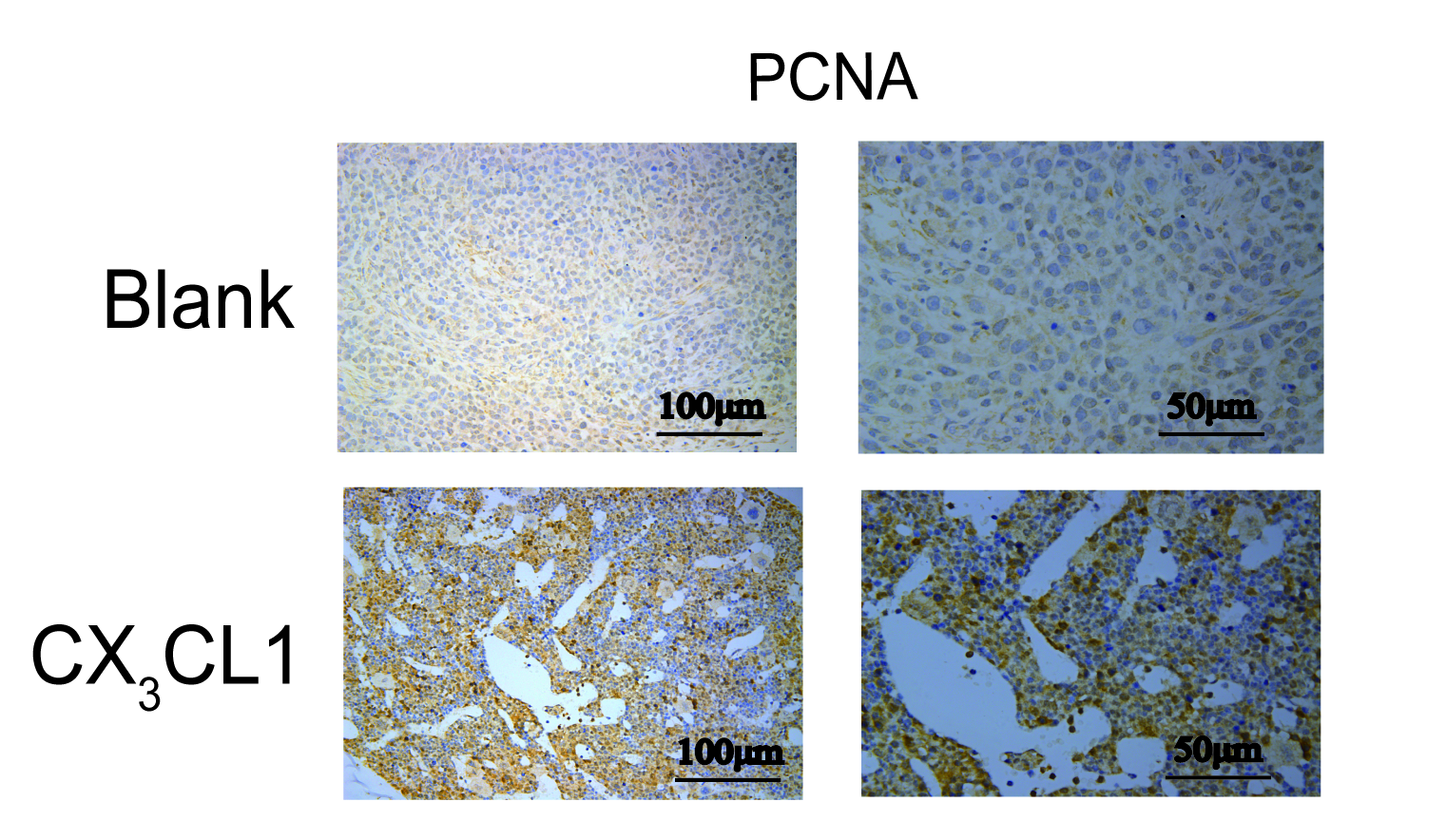

Supplement: Supplementary file 1 — Figure S1 [file JCMM-25-132-s001.tif]
